# A glutathione-based system for defense against carbonyl stress in *Haemophilus influenzae*

**DOI:** 10.1186/1471-2180-12-159

**Published:** 2012-07-31

**Authors:** Stephen P Kidd, Donald Jiang, Alexandra Tikhomirova, Michael P Jennings, Alastair G McEwan

**Affiliations:** 1 Research Centre for Infectious Disease, School of Molecular and Biomedical Science, The University of Adelaide, North Terrace Campus, Adelaide, 5005, Australia; 2 Institute for Glycomics, Griffith University (Gold Coast Campus), Parklands Drive, Southport, Queensland, 4215, Australia; 3 School of Chemistry and Molecular Biosciences, University of Queensland, Brisbane, Queensland, 4072, Australia

**Keywords:** Stress response, *H. influenzae*, Reactive aldehydes

## Abstract

**Background:**

*adhC* from *Haemophilus influenzae* encodes a glutathione-dependent alcohol dehydrogenase that has previously been shown to be required for protection against killing by *S*-nitrosoglutathione (GSNO). This group of enzymes is known in other systems to be able to utilize substrates that form adducts with glutathione, such as aldehydes.

**Results:**

Here, we show that expression of *adhC* is maximally induced under conditions of high oxygen tension as well as specifically with glucose as a carbon source. *adhC* could also be induced in response to formaldehyde but not GSNO. An *adhC* mutant was more susceptible than wild-type *Haemophilus influenzae* Rd KW20 to killing by various short chain aliphatic aldehydes, all of which can be generated endogenously during cell metabolism but are also produced by the host as part of the innate immune response.

**Conclusions:**

These results indicate that AdhC plays a role in defense against endogenously generated reactive carbonyl electrophiles in *Haemophilus influenzae* and may also play a role in defense against the host innate immune system.

## Background

*Haemophilus influenzae* is a γ-Proteobacterium adapted to the human host. It exists as a commensal in up to 80% of the healthy population. It survives in the nasopharnyx, and can spread to other sites within the body and cause disease [1]. *H. influenzae* requires a number of exogenous cofactors for growth including cysteine for the production of glutathione (GSH) [2]. In addition to its role in defence against oxidative stress [2,3] GSH forms adducts with toxic electrophilic molecules. Glutathione-dependent alcohol dehydrogenase (AdhC) catalyses the NAD^+^-dependent oxidation of a GSH-formaldehyde adduct [4,5]. Expression of *adhC* in a variety of bacteria is associated with defense against formaldehyde stress and is correspondingly regulated in the response to the presence of formaldehyde [6]. It is also established that AdhC catalyses the NADH-dependent reduction of *S*-nitrosoglutathione (GSNO), a molecule generated during the conditions of nitrosative stress that occurs in human cells in response to invading pathogens such as *H. influenzae*. Unlike other aldehyde dehydrogenase enzymes AdhC cannot use ethanol or formaldehyde directly, but uses the adducts which spontaneously form with GSH (hence the nomenclature, GSH-dependent formaldehyde dehydrogenase) [7]. AdhC from different sources is known to catalyse the concurrent oxidation of formaldehyde and reduction of GSNO [8,9]. We have previously observed that AdhC of *H. influenzae* does function in GSNO metabolism [10].

*H. influenzae* does not use methanol as a carbon source (the by-product of which is formaldehyde) and cannot assimilate formaldehyde. Therefore, a source of formaldehyde substrate for AdhC from the host environment is not obvious; however, bacteria do encounter a variety of aldehydes. Neutrophils use myeloperoxidase to produce glycoaldehyde and acrolein at sites of inflammation while short chain sugar aldehydes such as glyceraldehyde 3-P and erythrose 4-P are produced endogenously as intermediates of the bacterial metabolism of sugars [11]. All these short chain aldose sugars mentioned can undergo auto-oxidation to more toxic dicarbonyl species [12]. In this paper we report the effect of reactive carbonyl species on growth of *H. influenzae*. This provides a new insight into the physiological role of AdhC in non-methylotrophic bacteria.

## Methods

### Bacterial strains and growth conditions

*H. influenzae* strains were cultured on Brain heart infusion (BHI) medium or chemically defined media (CDM). BHI was prepared with 3.7% (wt/vol) BHI Powder (Oxoid). For solid medium, 1.5% (wt/vol) agar powder was added. Medium was sterilized by autoclaving at 121°C for 20 min. Levinthal blood (10% [wt/vol]) was added for solid medium. BHI broth required NAD (2 μg/ml) and 10 μg/ml hemin solution (0.1% [wt/vol] hemin, 0.1% [wt/vol] L-histidine, 4% [vol/vol] triethanolamine). Solutions for media were sterilized individually, either by filter sterilizing or by autoclaving. The solutions were mixed under sterile conditions. CDM was prepared mostly as described by Coleman *et al.*[13]. The exception to this protocol is the use of RPMI 1640 without glucose (Invitrogen) and the addition of 0.4% of the appropriate sugar or carbon source. In standard procedures the final pH of CDM was adjusted to 7.56 by NaHCO_3_. CDM was sterilized by filter sterilization through a 0.22-μm filter.

### Reverse transcriptase PCR

RNA was extracted from *H. influenzae* Rd KW20 at the time points 3 h, 5.5 h and 8 h during growth cycle by using a QIAGEN RNeasy minikit (QIAGEN). RNA was quantified using an A_260_ reading and then checked for DNA contamination by PCR; no product was detected. RNA was further treated to remove any residual DNA by using Promega DNase (Promega). The reverse transcriptase (RT) reaction was performed using a QIAGEN Omniscript reverse transcriptase kit. The products of this reaction were used in a multiplex PCR with primers for the 16 S rRNA gene:

16SFOR: 5’-AGTCCACGCCCTAAACGATGT-3’ and

16SREV: 5’-TACTCCCCAGGCGGTCAAT-3’; and primers from *estD* to *adhC*:

Est1: 5’-CCCAAGGCTGCTCGGTC-3’ and

Adh1, 5’-TTCAACGCGTCCGTTCCAA-3’.

PCR was carried out with New England Biolabs Taq polymerase using an initial 96°C for 10 min followed by 30 cycles of 96°C for 45 s, 54°C for 45 s, and 72°C for 30 s and a final elongation step of 72°C for 10 min.

### Growth assays

Cells were cultured in rich media (BHI, Oxoid UK) or chemically defined media (CDM). Unless otherwise stated, analysis of the growth of *H. influenzae* strains was carried out using CDM. For rich media cells were grown on BHI medium supplemented with NAD (2 μg/ml) and 10 μg/ml hemin solution. Overnight growth cultures were inoculated into 5 ml of media and grown until log phase prior to the assay. The OD_600_ of the cultures were measured (Hitachi U-3000 spectrophotometer) to standardize the amount of cells inoculated. Routinely 1 ml was inoculated into 50 ml of CDM in a 250-ml conical flask. For analysis of the effects of oxygen supply to the cells, cultures were grown in 250 ml conical flasks with 25 ml, 75 ml and 150 ml medium. This has been previously used and shown to provide the oxygen transfer coefficents (kLa) values of 87.4 h^-1^ (high), 27.8 h^-1^ (medium) and 11.5 h^-1^ (low) respectively [14,15].

Different specific concentrations of stress agent were added to the medium. Cultures were incubated aerobically at 37°C with shaking at 190 rpm. OD_600_ measurements were taken at different time points for 10 h. The assays were done in triplicate. Assay results were represented as growth curves over this period or, for clarity for the large set of clinical isolates, as percentages of survival at this time point.

### GSNO reductase enzyme assays

NADH-dependent GSNO reductase activity was measured as previously described [10]. Fresh overnight cultures of *H. influenzae* were inoculated into 100 ml of CDM in 500 ml conical flasks and grown aerobically at 37°C with shaking at 190 rpm until an OD_600_ measurement between 0.4 and 0.6 was obtained. The cells were harvested (5,000 × g at 4°C for 10 min) and washed twice with 0.1 M phosphate buffer (pH 7.0) before resuspending in 2 ml of phosphate buffer. The suspension was frozen at −80°C, thawed at room temperature, given a brief vortexing, and frozen again at −80°C. This freeze-thaw process was performed four more times before the cells were centrifuged at 13,000 × g at 4°C for 15 min. The final supernatant (cell extract) was used for assays. The total protein concentration of the supernatant was determined spectrophotometrically using the formula protein (mg/ml) (1.55 × A_280_) – (0.76 × A_260_) ^19^. GSNO reductase activity was expressed as μmol of NADH oxidized per minute per mg of total protein. The assays were done in triplicate.

## Results

### AdhC is expressed under aerobic conditions and required for aerobic growth in *H. influenzae*

**Figure 1:**
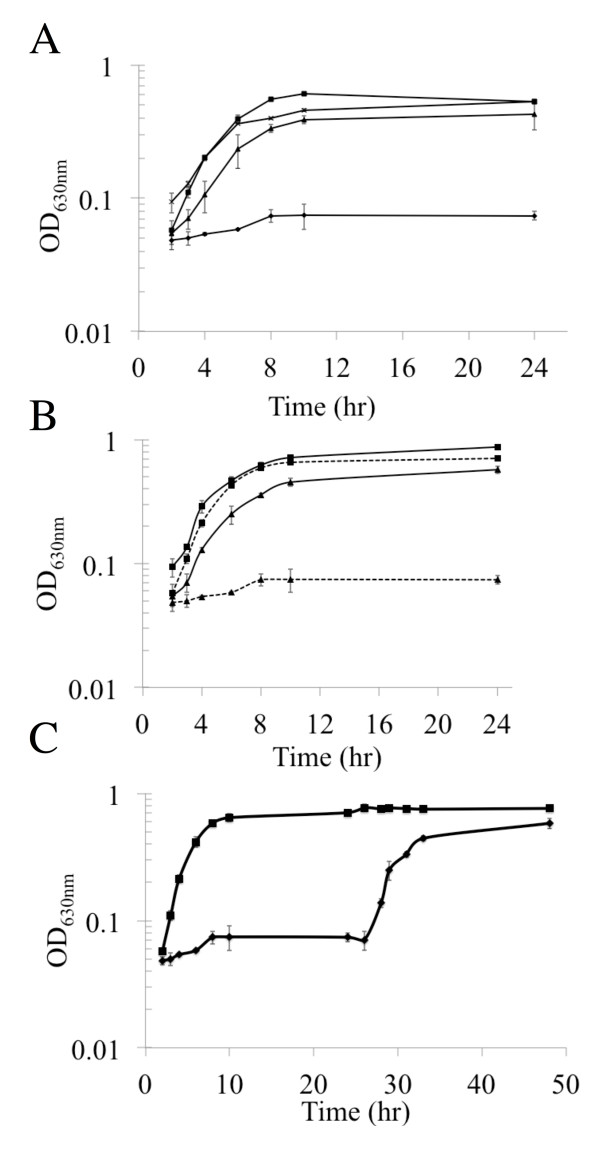
**AdhC in *H. influenzae *is required for growth with glucose at high oxygen.(A)** The Rd KW20 wild type and *adhC* mutant were grown in CDM at low oxygen tension (**×**, RdKW20 and ▲ , *adhC* mutant) and then high oxygen tension (□, RdKW20 and ♦, *adhC* mutant). **(B)** As a comparison the Rd KW20 was grown in BHI (■) and CDM (■) and the *adhC* mutant was grown in BHI (▲) and CDM (▲ with dotted lines). **(C)** Rd KW20 (■) and *adh*C mutant (♦) were then grown with high oxygen until 24 hr when the oxygen tension was changed to low oxygen.

We have previously observed that an *adhC* mutant of *H. influenzae* Rd KW20 appeared to have a reduced growth under aerobic conditions compared to its wild-type strain [10]. To further characterize this altered phenotype and determine its direct link to aerobic growth pathways and oxygen, we performed various growth assays using established parameters for low, medium and high levels of aeration to correlate to oxygen levels. We also used rich media and chemically defined media (providing only glucose as the carbon source) (Figure 1A and 1B). At high oxygen levels and in CDM the *adhC* mutant did not grow. Both wild type and *adhC* mutant cells were then grown at high oxygen for 24 h before being directly transferred to low oxygen conditions for a further 20 h (Figure 1C). Upon the switch in oxygen tension the *adhC* mutant cells grew.

**Figure 2:**
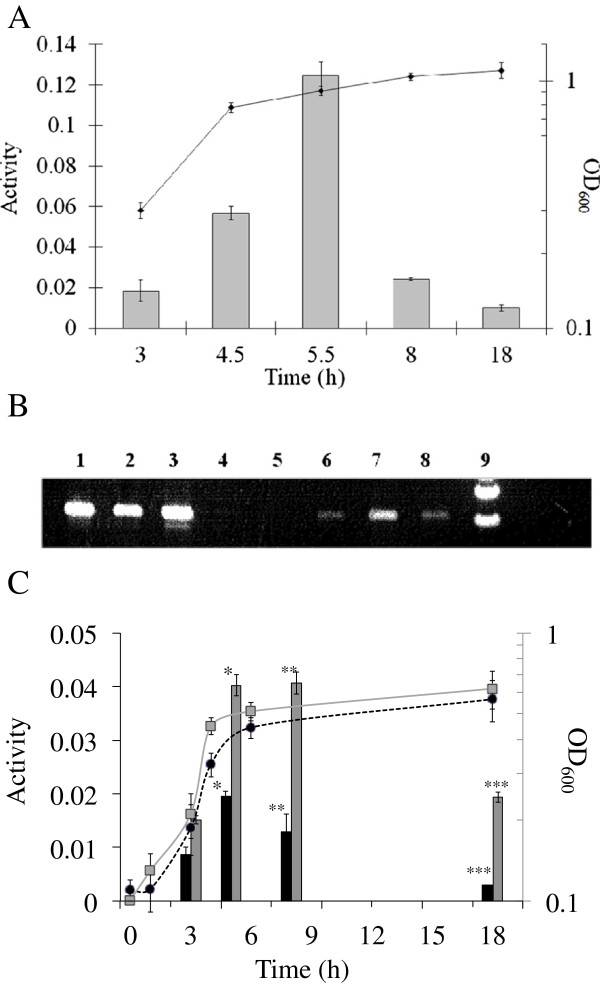
**Change in AdhC specific activity during growth of *H. influenzae*.(A)** Samples were taken and assayed for AdhC enzyme activity from early log phase (3 hr), mid-log phase (4.5 h), log phase (5.5 h) late log phase (8 h) and stationary phase (18 h). **(B)** RT-PCR for the 16SrDNA (lanes 1–4) and *adhC*-*estD* (lanes 5–8) using RNA from the time points 3 h (lanes 1 and 6), 5.5 h (lanes 2 and 7) and 8 h (lanes 3 and 8). Lanes 4 and 5 are representative negative controls. Lane 9 is the ladder. **(C)** At time points throughout the *H. influenzae* growth phase AdhC specific activity was measured from cells grown with different oxygen tensions (low tension are the black bar and high oxygen tension are the grey bars). The enzyme activity is presented as change in NADH consumed per minute per mg total protein. Y-error bars indicate +/− 1 standard deviation of the mean. Units are μmol NADH oxidized min^-1^ mg protein^-1^. The growth curves are indicated by the OD_600_ of cells grown at low oxygen levels (black circle) and high oxygen levels (gray box). (*P < 0.001, **P < 0.002, ***P < 0.0005).

To assess whether AdhC was being expressed under these aerobic conditions in the wild type cells we firstly monitored AdhC activity during the growth cycle. The cells were assayed for AdhC activity (by assay of GSNO reductase activity), at different time points through the growth cycle. Figure 2A shows that AdhC activity increases during exponential phase, and then decreases in late exponential and stationary phase. RNA was also extracted from *H. influenzae* wild-type strain at early, mid and late log phase and RT-PCR was performed using 16 S and *adhC*-*estD* primers (Figure 2B). We also investigated the effect of differences in oxygen tension on AdhC expression by growing cultures in low, medium and high oxygen levels; Figure 2C shows that AdhC activity was highest in cells grown at highest oxygen tension and activity decreased as oxygen tension in the culture decreased. Taken together these results indicated that *adhC* expression in *H. influenzae* is highest under aerobic conditions and this is associated with glucose metabolism.

### AdhC is required for defense against reactive aldehydes

**Figure 3:**
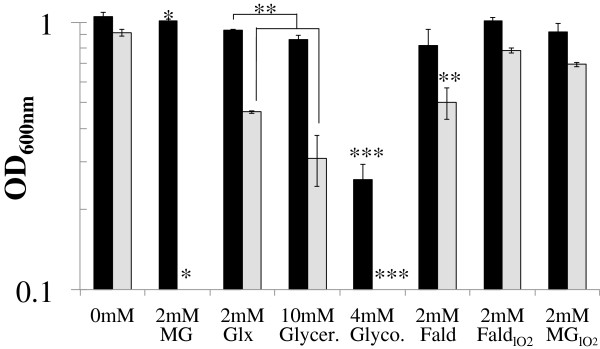
**Sensitivity of *H. influenzae adhC *strain to reactive aldehydes.** Wild type (Rd KW20; black bars) and the *adhC* mutant (grey bars) strains were grown in BHI media in the presence of increasing concentrations of particular reactive aldehydes with medium levels of oxygen (50 ml culture in 250 ml flask). The ability to resist the toxicity of these chemicals was measured by an OD_600_ reading after 18 h of growth. (*P < 0.0001, **P < 0.005, ***P < 0.0001). MG: methylglyoxal, Glx: glyoxal, Glycer: glyceraldehyde, Glyco: glycolaldehyde, Fald: formaldehyde, Fald_lO2_: formaldehyde with low oxygen, MG_lO2_, methylglyoxal with low oxygen.

To determine whether AdhC had a role in protection against the reactive aldehydes known to be relevant and toxic during aerobic growth, we grew wild-type (Rd KW20) and its isogenic *adhC* mutant in the presence of some of these compounds and measured the end point of growth (OD_600_), growth of any culture did not continue beyond the 18 hr point. Figure 3 shows that the *adhC* strain was more sensitive than wild-type to methylglyoxal; 2 mM methylglyoxyl completely inhibited the growth of the *adhC* mutant but had very little effect on growth of wild-type *H. influenzae* Rd KW20. Glyceraldehyde, glycolaldehyde and glyoxal also inhibited growth of the *adhC* mutant compared to wild-type *H. influenzae* Rd KW20. The overall growth profiles (lag phase and growth rates) were equally reduced in the *adhC* mutant compared to wild type. It has been demonstrated that the toxicity of short chain sugars, such as glyceraldehyde and glycolaldehyde, arises from the oxidation of their *ene*-diol tautomeric form which results in the formation of highly toxic dicarbonyl species [12]. If failure to protect against toxic dicarbonyl species underpinned the increased toxicity of reactive aldehydes towards the *adhC* mutant, then it ought to be possible to rescue such mutants using 1 mM 1,2-diaminobenzene (DAB) a compound that quenches the toxicity of dicarbonyl species. The addition of DAB did partially restore the growth of the *adhC* mutant in the presence of glycolaldehyde (Table 1). Consistent with this, under conditions of low oxygen where the toxic effect of these molecules is reduced, the susceptibility of the *adhC* mutant to these aldehydes is reduced (Figure 3). Given that previous studies on bacterial AdhC enzymes have focussed on its role in formaldehyde detoxification, we also assayed for formaldehyde sensitivity in the *H. influenzae adhC* mutant. The *adhC* mutant was slightly more sensitive than wild type to formaldehyde under high oxygen conditions when cultured in CDM, but was not at all under low conditions (Figure 3).

**Table 1:** **The growth rates of Rd KW20 and *adhC*; with 2 mM glycolaldehyde and 1 mM 1,2-diaminobenzene (DAB)**

**Strains**	**Growth rate (doubling per hour)**
Rd KW20	1.10 ± 0.14
Rd KW20 + glycolaldehyde	0.80 ± 0.37
Rd KW20 + glycol. + DAB	1.47 ± 0.35
*adhC*	0.79 ± 0.34
*adhC* + glycolaldehyde	0.20 ± 0.10
*adhC* + glycol. + DAB	0.51 ± 0.27

### AdhC is induced by formaldehyde but not by GSNO

**Figure 4:**
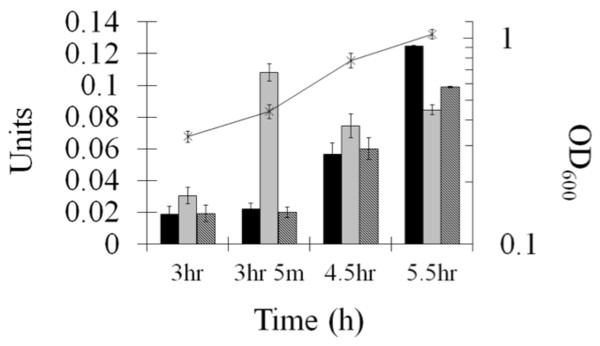
**Induction of AdhC activity by formaldehyde.** The activity of AdhC (as a measure of the change in NADH consumed per minute per mg total protein as described in the Materials and methods) was determined at time points in cells grown in BHI media alone (black bars) and then in media with 0.3% formaldehyde added at 3 h (light grey bars) and with 1 mM GSNO added (dark grey bars).

To determine whether the NmlR system, which controls AdhC expression, responded to nitrosative stress we investigated the effect of GSNO on AdhC activity. There was no change in AdhC activity upon addition of GSNO (the Units of activity remained at the same level as none added; 0.02 ± 0.005 μmol of NADH oxidized per minute per mg of total protein), suggesting that NmlR_HI_ in *H. influenzae* does not respond to nitrosative stress (Figure 4). As previously investigated, *Escherichia coli* and *H. influenzae* cells grown with formaldehyde had higher AdhC activity [16]; we tested a range of reactive aldehydes to ascertain whether they could induce *adhC* expression in *H. influenzae*. Figure 4 shows that addition of formaldehyde to *H. influenzae* caused a 5-fold rise in AdhC activity 5 minutes after its addition. AdhC activity was not induced by methylglyoxyl and glycolaldehyde under the same conditions (in both cases the Units of activity remained at the same level as with no chemical added; 0.02 ± 0.005 μmol of NADH oxidized per minute per mg of total protein).

## Discussion

The expression of *adhC* is regulated by the MerR family transcription factor NmlR_HI_[10]. Regulators of this family generally function as both weak repressors, and as activators when in the presence of their cognate stress effector. We have previously reported that expression of GSNO reductase activity in *H. influenzae* requires both *adhC*, the structural gene encoding the enzyme activity, as well as its regulator *nmlR*_HI_ under growth conditions with no exogenous stress. Mutant strains of *H. influenzae* in which the *adhC* or *nmlR*_HI_ genes have been inactivated do not express detectable GSNO reductase activity [10]. A reasonable conclusion was that under these conditions NmlR_HI_ is in its activator conformation and therefore endogenously generated molecules are the cognate “stress” for which it responds. Attempts to identify the cognate ligand or the environmental stimuli, which acts to switch NmlR_HI_, to an activator form have been unsuccessful.

In mammalian systems AdhC functions in detoxification of a range of reactive aldehyde species as well as in defense against GSNO. Our results suggest that there may be a similar role for AdhC in *H. influenzae*. Glycoaldehyde is produced from serine by the action of myeloperoxidase [17]. This is one of several types of reactive aldehydes that are produced by activated neutrophils at sites of inflammation. The toxicity of glycoaldehyde arises from the oxidation of its *ene*-diol tautomer to form a highly reactive α, β-dicarbonyl species. This reaction requires oxygen or superoxide, consistent with AdhC activity being highest with increased oxygen levels and during the highest periods of metabolic reactions. Our observations are also consistent with previous *in silico* analyses analysis of gene expression in *H. influenzae* which showed that *adhC* expression was highest under aerobic conditions during which glucose would be metabolised mainly via the pentose phosphate pathway [18,19]. Our data is consistent with these results as AdhC was required for growth with glucose as the carbon source under high oxygen culture conditions (Figures 1 and 2). Glyceraldehyde 3-phosphate and erythrose 4-phosphate are both intermediates in this pathway. It has been noted that the equilibrium constant for the aldolase reaction means that in glycolysis the concentration of glyceraldehyde 3-phosphate is kept very low. This may not be the case when the pentose phosphate pathway is the dominant glucose oxidation pathway that occurs under conditions of high oxygen tension [18,19].

Recently, it is has been observed that an NmlR homologue in *Bacillus subtilis* (AdhR) activates gene expression in response to methylglyoxyl and formaldehyde [20]. One cysteine (C54) was shown to be required for activation of gene expression and this led Antelmann and co-workers [20] to propose that *Bacillus* AdhR is activated by *S*-alkylation of this cysteine residue. AdhR contains a single conserved cysteine, as in the NmlR_sp_ transcription factor from *Streptococcus pneumoniae*[21]. In *H. influenzae* we only observed induction of *adhC* by NmlR_HI_ upon addition of formaldehyde. In contrast to the situation in *B. subtilis* and *S. pneumoniae*, NmlR_HI_ possesses three conserved cysteine residues and is closely related to the NmlR regulators from *Neisseria* species [22]. Thus, there may be significant differences in the mechanism of the sensing of reactive carbonyl compounds by transcription factors of the NmlR family.

## Conclusions

Uniquely, *H. influenzae* utilizes an AdhC enzyme for the concurrent roles of protection against an exogenous stress (GSNO) as well as the endogenously generated and harmful reactive aldehydes. AdhC is essential for *H. influenzae* growth under conditions of high oxygen and with glucose as the carbon source. This role is through the detoxification of different reactive carbonyl compounds.

## Competing interests

The authors declare that they have no competing interests.

## Authors’ contributions

SPK helped in the design of the study, participated in the growth studies, the enzyme assays and the RT-PCR experiments and, helped draft the manuscript. DJ and AT participated in the growth studies. MPJ and AGM were part of the design and conception of the study and the analysis of the data and writing the manuscript. All authors read and approved the final manuscript.
